# Causal Relationships of General and Abdominal Adiposity on Osteoarthritis: A Two-Sample Mendelian Randomization Study

**DOI:** 10.3390/jcm12010320

**Published:** 2022-12-31

**Authors:** Leifeng Lyu, Yuanqing Cai, Mofan Xiao, Jialin Liang, Guangyang Zhang, Zhaopu Jing, Rupeng Zhang, Xiaoqian Dang

**Affiliations:** 1 Department of Orthopaedics, The Second Affiliated Hospital of Xi’an Jiaotong University, NO. 157, Xiwu Road, Xi’an 710004, China; 2 Department of Gastroenterology, The First Affiliated Hospital of Xi’an Jiaotong University, Xi’an 710061, China

**Keywords:** body mass index, waist circumference, hip circumference, waist-to-hip ratio, osteoarthritis, mendelian randomization

## Abstract

Background: Adiposity is closely related to osteoarthritis, but the causal effects of different types of adiposity on osteoarthritis are indistinct. This study conducted a Mendelian Randomization (MR) analysis for the causal effects of general adiposity and abdominal adiposity on knee osteoarthritis (KOA) and hip osteoarthritis (HOA). Methods: The general adiposity was assessed by body mass index (BMI), while the abdominal adiposity was evaluated with waist circumference (WC), hip circumference (HC), and waist-to-hip ratio (WHR). The data used in this two-sample MR analysis originated from genome-wide association studies (GWAS). Significant (*p* < 5 × 10^−8^) and independent (r^2^ < 0.01) single-nucleotide polymorphisms were selected as instrumental variables for the MR analysis. Subsequently, this study used the inverse variance weighted, weighted median, and other methods for the causal inference, and the results were presented as odds ratios (OR). Moreover, sensitivity analyses were conducted to assess the stability and reliability of the results. Results: The MR results revealed positive causal effects of BMI on KOA (OR: 1.694; 95% CI: from 1.492 to 1.923; *p* = 3.96 × 10^−16^) and HOA (OR: 1.412; 95% CI: from 1.196 to 1.666; *p* = 4.58 × 10^−5^). Additionally, WC and HC both positively and causally related to KOA (WC: OR: 1.827; 95% CI: from 1.564 to 2.134; *p* = 2.68 × 10^−14^; HC: OR: 1.610; 95% CI: from 1.357 to 1.912; *p* = 5.03 × 10^−8^) and HOA (WC: OR: 1.491; 95% CI: from 1.254 to 1.772; *p* = 5.85 × 10^−6^; HC: OR: 1.439; 95% CI: from 1.205 to 1.719; *p* = 5.82 × 10^−5^). However, no causal relationship existed between WHR and obesity. These results were robust according to the sensitivity analyses. Conclusions: This study indicated that both general and abdominal obesity had positive causal effects on knee osteoarthritis and hip osteoarthritis.

## 1. Introduction

Osteoarthritis (OA) is the most common degenerative disease of joints involving a complex process composed of inflammatory and metabolic factors, which might lead to loss of articular cartilage, synovitis, subchondral bone sclerosis, and osteophyte formation [1,2]. Knee osteoarthritis (KOA) and hip osteoarthritis (HOA) are the most common types of OA. It is estimated that KOA and HOA are the 11th highest contributors to global disability and the 4th in aging populations [3]. Even though a large number of scholars are increasingly focused on OA, the pathogenesis of OA is still unclear. The risk factors, including obesity, inflammation, bone metabolism, and muscle strength, could affect the incidence of osteoarthritis [4]. According to the former study, nearly 30% of over 45 years old individuals had radiographic evidence of KOA, and almost half of them had knee symptoms, which brought a large burden on society [5].

Obesity is defined as excessive fat accumulation that might impair health and is diagnosed at a body mass index (BMI) ≥ 30 kg/m^2^ [6]. The prevalence of obesity has increased worldwide to pandemic proportions in the past 50 years and caused plenty of problems [7]. A cohort study showed that obesity could increase the risk of hand, hip, and knee OA and contained a dose-response gradient with increasing BMI [8]. Devyani et al. also found obesity was associated with knee OA risk, and it could promote inflammatory processes implicated in the pathogenesis of OA so that the prevalence of OA increased with obesity [2,9]. Generally speaking, it might be inaccurate to evaluate obesity just by BMI levels due to the existence of abdominal obesity. The evidence showed that combining the BMI and body shape anthropometric traits such as waist circumference (WC), hip circumference (HC), and waist-to-hip ratio (WHR) could be more effective when assessing obesity [10]. However, the existing studies did not combine BMI and body shape traits to judge the level of obesity and could not identify the causality between obesity and OA comprehensively.

Mendelian randomization (MR) analysis is a powerful method that takes genetic variants as instrumental variables (IVs) to assess the causality between exposures and outcomes by imitating the randomized controlled trial [11,12]. Since genotypes appear before the occur of disease and are largely unrelated to lifestyle or environmental factors after birth, the MR method could minimize the confounders and avoid reverse causality [13]. Thus, MR has been widely used in causal inference in various research. In this study, single-nucleotide polymorphisms (SNPs) were chosen as the IVs to perform a bidirectional MR analysis without causal relationships between obesity (BMI, WC, HC, and WHR) and osteoarthritis (KOA and HOA).

## 2. Materials and Methods

### 2.1. Study Design

**Figure 1:**
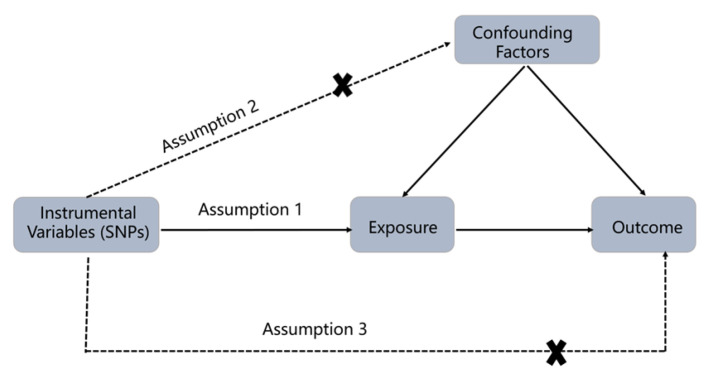
The schematic diagram of Mendelian Randomization (MR). Three assumptions should be met: Assumption 1: The SNPs should be closely related to exposures; Assumption 2: The SNPs selected should be independent of confounders; Assumption 3: The SNPs should affect results only through exposure but not the direct correlation. (SNPs: single nucleotide polymorphisms).

The study design is shown in Figure 1. Three assumptions should be met when performing MR analysis. Assumption 1. The SNPs should be closely related to exposures. Assumption 2. The SNPs selected should be independent of confounders. Assumption 3. The SNPs should affect results only through exposure but not the direct correlation. As mentioned earlier, this study took the SNPs as to IVs to conduct a bidirectional two-sample MR analysis to evaluate the causal relationships between BMI, WC, HC, WHR, and osteoarthritis.

### 2.2. Data Source

The data used in MR analysis came from the genome-wide association studies (GWAS) datasets. The selection of BMI-associated genetic predictors originated from the GIANT Consortium based on GWAS with 322,154 individuals and 2,554,668 SNPs of European ancestry [14]. Additionally, the genetic variants for WC and HC, respectively, brought 232,101 (2,565,408 SNPs) and 73,137 (2,738,303 SNPs) European individuals from GIANT Consortium into the study [15]. Moreover, WHR genetic predictors were also obtained from a published GWAS dataset of GIANT, which was icon h based on 118,003 European participants and 2,466,102 SNPs [15]. Genetic predictors of osteoarthritis were obtained from UK Biobank and arcOGEN resources, which contained 403,124 individuals for KOA (24,955 knee osteoarthritis cases and 378,169 controls) and 393,873 subjects for HOA (15,704 hip osteoarthritis cases and 378,169 controls). Previous researchers had elucidated the details of the data used for KOA and HOA [16]. The data about osteoarthritis were from European ancestry. All datasets are available at the publicly available GWAS datasets (https://gwas.mrcieu.ac.uk (accessed on 7 November 2022)).

### 2.3. Selection of Instrumental Variables

In this study, the SNPs were selected as the IVs to conduct a Two-sample MR analysis. Genome-wide significant (*p* < 5 × 10^−8^) SNPs were extracted as IVs. Then, the linkage disequilibrium (LD) was tested to ensure that the SNPs were independent (r^2^ < 0.01) and that they would be excluded if containing linkage disequilibrium. All the SNPs that might be associated with the confounding factors were removed. Finally, the F-statistics were calculated, and weak IVs were excluded at *F* < 10.

### 2.4. Statistical Analysis

The inverse variance weighted (IVW), weighted median and MR-Egger were mainly used to assess the causal associations between BMI, WC, HC, WHR, and OA. The IVW method analyzes each Wald ratio and provides a consistent estimate of the causal effect when all instrumental variables are valid [17]. The weighted median method gives unbiased estimates even when up to 50% of the information comes from invalid instrumental variables [18]. The MR-Egger intercept was conducted to test horizontal pleiotropy. However, this study concentrated on the effect size rather than the statistical significance of MR-Egger, for the statistical power is low [19]. Simple mode and Weighted mode methods were also used to evaluate the causal relationships between obesity and osteoarthritis.

Additionally, Cochran’s *Q* test and I^2^ statistics were used to assess the heterogeneity. Then, we performed the MR-Egger intercept to test the pleiotropy. To evaluate the effectiveness and stability of MR results, this study then conducted the sensitivity analysis using the ‘leave-one-out’ sensitivity test.

All statistical analyses were performed by the ‘Two-Sample MR’ package in R (version 4.2.1) software. The results were considered statistically significant at *p* < 0.05. The additional ethical approval or consent to participate was not required in the research on account that the analysis was based on existing publications.

## 3. Results

### 3.1. Selected SNPs for This Study

Firstly, this study selected BMI and body shape anthropometric traits (WC, HC, and WHR) as risk factors to conduct the MR analysis on OA. Then osteoarthritis was taken as a risk factor, while obesity traits were treated as outcomes to perform MR analyses. The significant and independent SNPs were extracted (*p* < 5 × 10^−8^, r^2^ < 0.01), and the weak IVs were excluded (*F* < 10), then the rest SNPs were chosen for further MR analysis. The information on the selected SNPs used for MR analysis was listed in Supplementary Tables S1–S8. For the osteoarthritis outcome, there were 77 BMI-related SNPs with a mean of *F* = 55.94, 42 WC-related SNPs with a mean of *F* = 50.17, 51 HC-related SNPs with a mean of *F* = 47.98, and 20 WHR-related SNPs with a mean of *F* = 46.40 selected for MR analysis.

### 3.2. Causal Relationships between Body Mass Index and Osteoarthritis

**Figure 2:**
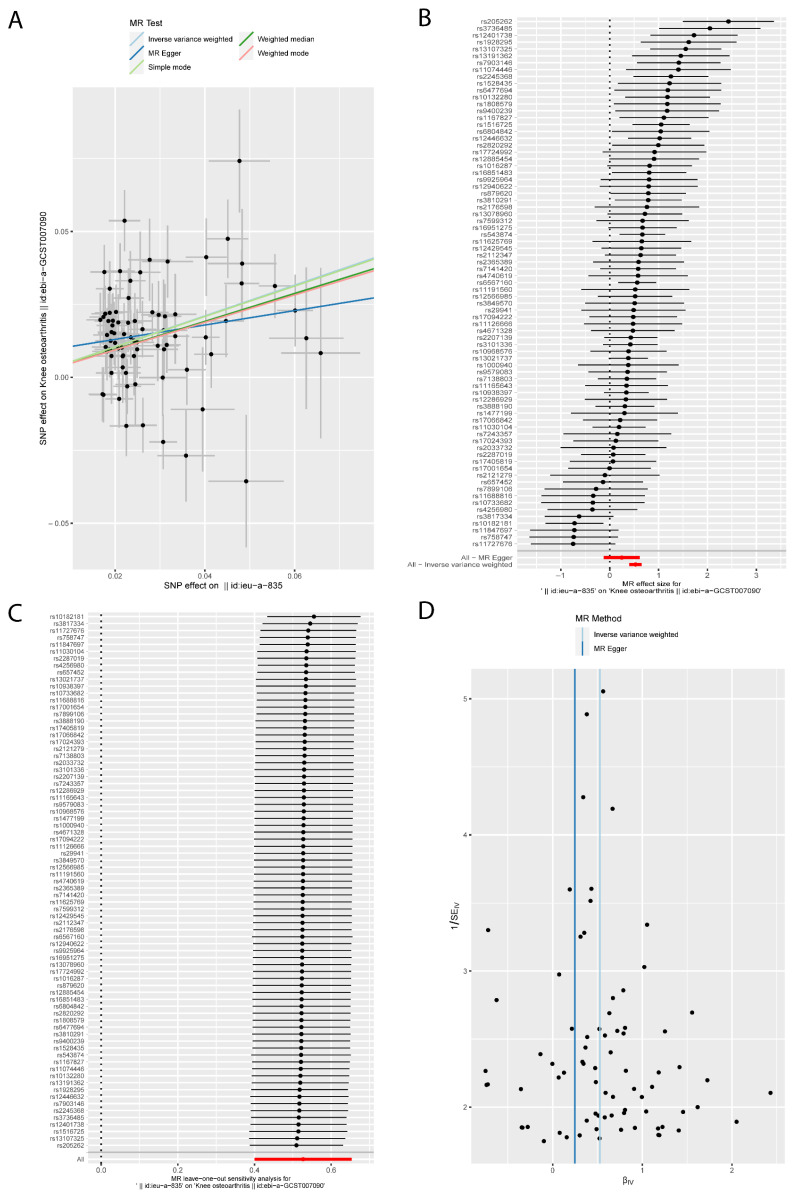
The MR results of body mass index on knee osteoarthritis. (**A**) Scatter plot about the causal effect of body mass index on knee osteoarthritis. (**B**) Forest plot for the overall causal effects of body mass index on knee osteoarthritis. (**C**) Leave-one-out analysis for the causal effect of body mass index on knee osteoarthritis. (**D**) Funnel plot of SNPs related to body mass index and knee osteoarthritis.

**Figure 3:**
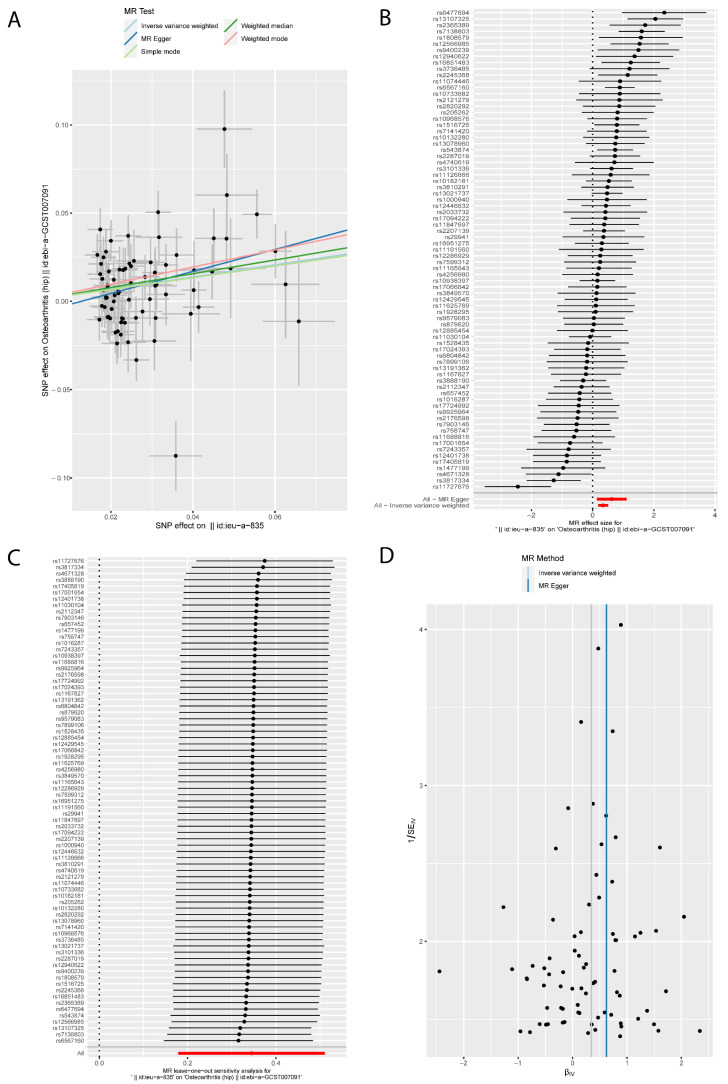
The MR results of body mass index on hip osteoarthritis. (**A**) Scatter plot about the causal effect of body mass index on hip osteoarthritis. (**B**) Forest plot for the overall causal effects of body mass index on hip osteoarthritis. (**C**) Leave-one-out analysis for the causal effect of body mass index on hip osteoarthritis. (**D**) Funnel plot of SNPs related to body mass index and hip osteoarthritis.

The MR results of BMI on KOA were listed in Table 1 and Figure 2, which were reported as odds ratios (OR). The IVW method showed that BMI had a positive causal effect on KOA (OR: 1.694; 95% CI: from 1.492 to 1.923; *p* = 3.96 × 10^−16^); similar results were obtained from the Weighted median (OR: 1.615; 95% CI: from 1.403 to 1.860; *p* = 2.57 × 10^−11^) method. According to Cochran’s Q, I^2^, and MR-Egger intercept test, there was heterogeneity (*Q* = 155.92, *p* = 1.88 × 10^−7^; I^2^ = 51.26%) but no pleiotropy (intercept = 0.008, *p* = 0.120) in the results. Then the ‘leave-one-out’ sensitivity indicated that the causal effect of BMI on KOA was not affected by individual SNPs (Figure 2C), which means the results were stable and reliable. Additionally, this study conducted MR analyses between BMI and HOA; the results are listed in Table 2 and Figure 3. With a 1 standard deviation (SD) increase in BMI, the risk of HOA increases to approximately 1.4–1.9 times based on IVW (OR: 1.412; 95% CI: from 1.196 to 1.666; *p* = 4.58 × 10^−5^), Weighted median (OR: 1.477; 95% CI: from 1.225 to 1.782; *p* = 4.54 × 10^−5^) and MR-Egger (OR: 1.864; 95% CI: from 1.144 to 3.035; *p* = 0.014). Cochran’s *Q* Statistic showed there was heterogeneity in the results (*Q* = 169.21, *p* = 4.73 × 10^−9^). The positive causal effect of BMI on HOA was not affected by the directional pleiotropy (intercept = −0.008, *p* = 0.239) or single SNP (Figure 3).

### 3.3. Causal Relationships between Waist Circumference and Osteoarthritis

**Figure 4:**
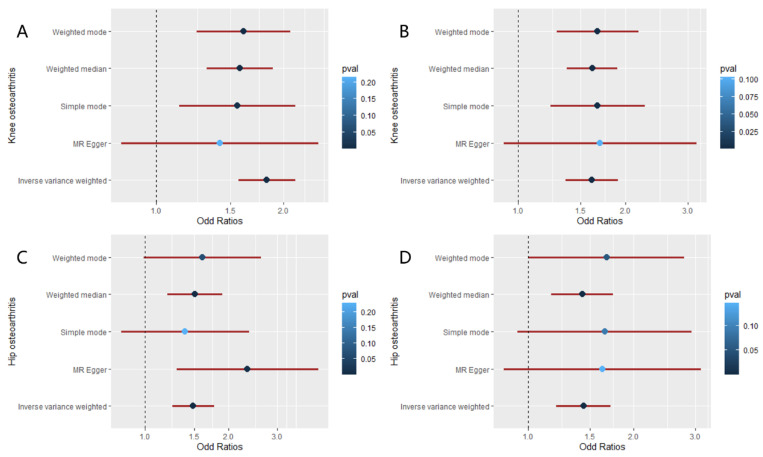
The forest plot about the causal effects of WC and HC on osteoarthritis. (**A**) Forest plot of the casual effect of WC on KOA. (**B**) Forest plot of the casual effect of WC on HOA. (**C**) Forest plot of the casual effect of HC on KOA. (**D**) Forest plot of the casual effect about HC on HOA. (WC: waist circumference; HC: hip circumference; KOA: knee osteoarthritis; HOA: hip osteoarthritis; OR: odds ratio).

Then the causal relationships between waist circumference and osteoarthritis were also analyzed. Table 1, Figure 4, and Figure S1 showed the results of the MR analysis of WC on KOA. The IVW (OR: 1.827; 95% CI: from 1.564 to 2.134; *p* = 2.68 × 10^−14^), Weighted median (OR: 1.576; 95% CI: from 1.315 to 1.889; *p* = 8.65 × 10^−7^) methods demonstrated that WC had a positive causal effect on KOA. There was heterogeneity (*Q* = 72.99, *p* = 0.002) but no pleiotropy (intercept = 0.007, *p* = 0.334), and the ‘leave-one-out’ analysis indicated that the results were stable and reliable (Figure S1C). Moreover, similar relationships were obtained based on the MR results about WC and HOA (Table 2, Figure 4 and Figure S2). The IVW method showed a positive causal effect of WC on HOA (OR: 1.491; 95% CI: from 1.254 to 1.772; *p* = 5.85 × 10^−6^). Weighted median (OR: 1.514; 95% CI: from 1.205 to 1.902; *p* = 3.7 × 10^−4^) and MR-Egger (OR: 2.343; 95% CI: from 1.301 to 4.221; *p* = 0.007) also supported the results. Cochran’s *Q* Statistic indicated the heterogeneity in the results (*Q* = 57.54, *p* = 0.045). No pleiotropy (intercept = −0.012, *p* = 0.124) or the SNPs that affected the results was observed (Figure S2C).

### 3.4. Causal Relationships between Hip Circumference and Osteoarthritis

As for the relationships between HC and KOA, the MR results are shown in Table 1, Figure 4, and Figure S3. The IVW (OR: 1.610; 95% CI: from 1.357 to 1.912; *p* = 5.03 × 10^−8^) and Weighted median (OR: 1.616; 95% CI: from 1.371 to 1.903; *p* = 9.78 × 10^−9^) indicated a positive causal effect of HC on KOA. There was heterogeneity (*Q* = 135.41, *p* = 4.96 × 10^−10^; I^2^ = 63.81%) but no pleiotropy (intercept = −0.002, *p* = 0.862) observed in the causal relationship. The ‘leave-one-out’ analysis revealed that the results were not affected by individual SNPs. Additionally, the study results concerning HC and HOA listed in Table 2, Figure 4, and Figure S4 demonstrated that HC had a positive causal relationship with HOA based on the IVW (OR: 1.439; 95% CI: from 1.205 to 1.719; *p* = 5.82 × 10^−5^) and Weighted median (OR: 1.428; 95% CI: from 1.164 to 1.752; *p* = 6.29 × 10^−4^). Heterogeneity existed in the causal effect between HC and HOA (*Q* = 92.43, *p* = 1.75 × 10^−4^). There was no pleiotropy (intercept = −0.004, *p* = 0.697) observed in the research. The ‘leave-one-out’ test in Figure S4C showed that no single SNP would influence the results.

### 3.5. Causal Relationships between Waist-to-Hip Ratio and Osteoarthritis

At last, we conducted the MR analyses between waist-to-hip ratio and osteoarthritis. The results of WHR and KOA are listed in Table 1. However, the MR analysis indicated a null causal effect of WHR on KOA by IVW (OR: 1.121; 95% CI: from 0.917 to 1.371; *p* = 0.264), Weighted median (OR: 1.088; 95% CI: from 0.878 to 1.350; *p* = 0.440), MR-Egger (OR: 1.103; 95% CI: 0.287 to 4.009; *p* = 0.918) and the other methods. Moreover, the causal associations between WHR and HOA were also assessed. According to the results in Table 2, the IVW (OR: 1.216; 95% CI: from 0.961 to 1.539; *p* = 0.103), Weighted median (OR: 1.128; 95% CI: from 0.869 to 1.465; *p* = 0.364) and MR-Egger (OR: 0.598; 95% CI: from 0.132 to 2.704; *p* = 0.513) all detected a no causal effect of WHR on HOA. In total, there was no causal relationship between the waist-to-hip ratio and osteoarthritis.

## 4. Discussion

This study aimed to explore the relationships between obesity and osteoarthritis with a two-sample Mendelian Randomization analysis. It might be the first study to combine body mass index and body shape trait (WC, HC, and WHR) to assess the causal effects of obesity on osteoarthritis with the help of GWAS datasets. This study provided strong genetic evidence that BMI, WC, and HC, but not WHR, had positive causal effects on both knee and hip osteoarthritis.

Obesity has increased worldwide and caused plenty of problems with the development of society. At present, 39% of the population around the world is obese or overweight despite decades of efforts to control it [6]. Osteoarthritis is a global disease that causes great pain and dysfunction in the elderly, the incidence of which is rising annually. According to previous research, up to two-thirds of the elderly obese population was affected by OA [2]. Thus, it attracts plenty of researchers to explore the relationships between obesity and osteoarthritis. The body mass index is the most frequent index of general obesity, which is easily accessible and has clear categories [20]. Carlen Reyes et al. found that overweight and obesity could increase the risk of hand, hip, and knee OA with a dose–response gradient with increasing BMI through a cohort study in Spanish [8] and a 5% to 10% weight loss in the obese population could significantly improve osteoarthritis pain [21]. Another study also showed that weight loss might benefit osteoarthritis; it concluded that weight loss of 10–19.9% of baseline body weight had substantial clinical and mechanistic benefits compared with less weight loss, which would reduce pain and improve function. The results obtained by these above studies were consistent with our research.

In recent years, scholars have come to realize that abdominal obesity plays an important role in geriatric diseases. Nevertheless, the BMI could not distinguish between abdominal or peripheral fat despite its important role in assessing obesity [22]. On account of this, WHO promoted consultations and research related to abdominal obesity indices such as WC and WHR to compensate for the limitations of BMI [23]. This study studied the relationships concerning not only general obesity (BMI) but abdominal obesity (WC, HC, and WHR) with osteoarthritis, which provided a reference at which obesity measures could predict the risk of osteoarthritis. A cohort study conducted by Badley found that both BMI and WHR showed apparent associations between obesity and OA [24]. It was proved that being overweight earlier in adult life increased the risks of knee OA and hip OA based on the index of BMI, WC, HC, and WHR [25]. Christiansen et al. performed an observational study and indicated that higher WC increased the risk of incident low physical function in OA patients [26]. Similar research revealed elevated WC was associated with a slightly higher risk of disability over time in OA patients [27]. Additionally, a cross-sectional study indicated that either BMI or WHR was a strong predictor of osteoarthritis, but WHR could not increase the predictive ability of BMI when predicting osteoarthritis risk [28]. However, the observational and cohort studies above were limited due to the small amount of sample size and could not decrease bias, confounding factors, and so on. More importantly, these studies could only figure out the correlations but not causal relationships between obesity and osteoarthritis. Thus, we conducted MR analyses to assess the causal relationships with a large sample size based on GWAS in this study. The results revealed causal effects of BMI, WC, and HC but not WHR on OA, while no causal association between osteoarthritis on obesity provided genetic evidence about the causal relationships between different types of obesity and osteoarthritis.

According to previous studies, obesity could risk knee or hip osteoarthritis through mechanical factors for their load-bearing function. The main pathological features of OA patients with obesity are horizontal fracture of the osteochondral interface, cartilage erosion with chronic inflammation, and microvascular rupture [29]. Changes in load are related to the inflammatory state of articular cartilage and metabolic imbalance of biosynthesis, which ultimately leads to cartilage rupture [30]. It has shown that mechanical stress on extracellular matrix molecules or inflammatory cytokines would lead to the activation of hypertrophic chondrocytes [31], and both cartilage and subchondral bone are affected by mechanical stress. Additionally, some researchers also found that the BMI could interact with the misalignment of the knees to promote the progression of OA, and both the varus- and valgus-aligned knees had greater risks in the development of radiographic knee OA [32,33]. Obesity could also lead to bone marrow edema, which plays a critical role in the pathogenesis of knee osteoarthritis [34,35]. Based on these studies, there was no denying that mechanical stress played a crucial part in the occurrence of osteoarthritis. Apart from the mechanical factors, more and more evidence indicated that inflammation was also a crucial part between obesity and osteoarthritis [36]. According to the previous study, adipose tissues could produce pro-inflammatory cytokines (including TNF-α, IL-6, and IL-1) and adipokine (including leptin and adiponectin) to regulate articular chondrocytes [37]. As the main adipokine secreted by adipose cells, leptin and its receptor are closely associated with the stage of OA [38]. A cross-sectional study found that the levels of leptin were significantly higher in the OA patients than in the control group [39]. Another analysis involving 6408 participants also indicated that leptin levels but not adiponectin were associated with OA and partially mediated the association between adiposity and osteoarthritis [40]. Moreover, Griffin et al. also revealed that leptin played an essential part in obesity-related OA, while the adiposity alone is not enough to cause knee OA [41]. All of these studies showed the great importance of leptin in the relationship between obesity and osteoarthritis, and further research is needed to clarify the potential mechanism.

This was the first research to explore the causal associations in genetics between general obesity as well as abdominal obesity and osteoarthritis with the MR analyses. The MR analysis could control the unmeasured confounders and reverse causality biases that existed in the observational studies. Additionally, the genetic variants (large sample size and robustly associated SNPs) were taken as IVs to imitate the design of RCTs, which gave our study sufficient power to detect causal effects between obesity and osteoarthritis with high precision. However, there were some limitations in this study. Firstly, this MR analysis is based on European ancestry; whether similar results would be obtained in other ancestries is unknown. Moreover, the data in the research came from public datasets, making it hard to conduct subgroup analysis in osteoarthritis (hand OA, lower lamb OA, and so on).

## 5. Conclusions

The Mendelian Randomization analysis provides strong evidence that obesity plays an important role in the occurrence of osteoarthritis. Both general obesity (BMI) and abdominal obesity (WC and HC) had positive causal effects on knee and hip osteoarthritis. The results could deepen our understanding of the inner relationship between obesity and OA at genetic levels and raise our awareness of losing weight within reasonable limits.

## Figures and Tables

**Table 1:** The Mendelian randomization analysis results with regard to causal effect of BMI, WC, HC, and WHR on Knee osteoarthritis.

Exposure	Methods	SNP (*n*)	OR	OR 95% CI	*p*-Value
BMI	MR Egger	77	1.280	0.884, 1.854	0.195
	Weighted median	77	1.615	1.403, 1.860	2.57 × 10^−11^
	Inverse variance weighted	77	1.694	1.492, 1.923	3.96 × 10^−16^
	Simple mode	77	1.684	1.251, 2.267	9.51 × 10^−4^
	Weighted mode	77	1.600	1.323, 1.937	6.81 × 10^−6^
WC	MR Egger	42	1.413	0.825, 2.420	0.215
	Weighted median	42	1.576	1.315, 1.889	8.65 × 10^−7^
	Inverse variance weighted	42	1.827	1.564, 2.134	2.68 × 10^−14^
	Simple mode	42	1.555	1.132, 2.136	0.009
	Weighted mode	42	1.610	1.247, 2.079	7.22 × 10^−4^
HC	MR Egger	51	1.699	0.910, 3.175	0.103
	Weighted median	51	1.616	1.371, 1.903	9.78 × 10^−9^
	Inverse variance weighted	51	1.610	1.357, 1.912	5.03 × 10^−8^
	Simple mode	51	1.673	1.231, 2.273	0.002
	Weighted mode	51	1.673	1.283, 2.181	3.96 × 10^−4^
WHR	MR Egger	20	1.073	0.287, 4.009	0.918
	Weighted median	20	1.088	0.878, 1.350	0.440
	Inverse variance weighted	20	1.121	0.917, 1.371	0.264
	Simple mode	20	1.085	0.663, 1.776	0.749
	Weighted mode	20	1.061	0.680, 1.654	0.797

BMI: body mass index; WC: waist circumference; HC: hip circumference; WHR: waist-to-hip ratio; SNP: single nucleotide polymorphism; OR: odds ratio; CI: confidence interval.

**Table 2:** The Mendelian randomization analysis results with regard to causal effect of BMI, WC, HC, and WHR on Hip osteoarthritis.

Exposure	Methods	SNP (*n*)	OR	OR 95% CI	*p*-Value
BMI	MR Egger	77	1.864	1.144, 3.035	0.014
	Weighted median	77	1.477	1.225, 1.782	4.54 × 10^−5^
	Inverse variance weighted	77	1.412	1.196, 1.666	4.58 × 10^−5^
	Simple mode	77	1.396	0.858, 2.270	0.183
	Weighted mode	77	1.625	1.157, 2.281	0.006
WC	MR Egger	42	2.343	1.301, 4.221	0.007
	Weighted median	42	1.514	1.205, 1.902	3.7 × 10^−4^
	Inverse variance weighted	42	1.491	1.254, 1.772	5.85 × 10^−6^
	Simple mode	42	1.392	0.818, 2.369	0.230
	Weighted mode	42	1.606	0.987, 2.615	0.064
HC	MR Egger	51	1.629	0.854, 3.111	0.145
	Weighted median	51	1.428	1.164, 1.752	6.29 × 10^−4^
	Inverse variance weighted	51	1.439	1.205, 1.719	5.82 × 10^−5^
	Simple mode	51	1.657	0.936, 2.933	0.089
	Weighted mode	51	1.675	1.007, 2.786	0.053
WHR	MR Egger	20	0.598	0.132, 2.704	0.513
	Weighted median	20	1.128	0.869, 1.465	0.364
	Inverse variance weighted	20	1.216	0.961, 1.539	0.103
	Simple mode	20	1.445	0.849, 2.460	0.191
	Weighted mode	20	1.025	0.630, 1.667	0.922

BMI: body mass index; WC: waist circumference; HC: hip circumference; WHR: waist-to-hip ratio; SNP: single nucleotide polymorphism; OR: odds ratio; CI: confidence interval.

## Data Availability

All the data used in this study are available at GWAS datasets (https://gwas.mrcieu.ac.uk (accessed on 7 November 2022)).

## Supplementary Materials

The following supporting information can be downloaded at: https://www.mdpi.com/article/10.3390/jcm12010320/s1, Table S1: The selected SNPs that are associated with body mass index and knee osteoarthritis. Table S2: The selected SNPs that are associated with body mass index and hip osteoarthritis. Table S3: The selected SNPs that are associated with waist circumference and knee osteoarthritis. Table S4: The selected SNPs that are associated with waist circumference and hip osteoarthritis. Table S5: The selected SNPs that are associated with hip circumference and knee osteoarthritis. Table S6: The selected SNPs that are associated with hip circumference and hip osteoarthritis. Table S7: The selected SNPs that are associated with waist-to-hip ratio and knee osteoarthritis. Table S8: The selected SNPs that are associated with waist-to-hip ratio and hip osteoarthritis. Figure S1: The MR results for causal effect regarding waist circumference on knee osteoarthritis. Figure S2: The MR results for causal effect regarding waist circumference on hip osteoarthritis. Figure S3: The MR results for causal effect regarding hip circumference on knee osteoarthritis. Figure S4: The MR results for causal effect regarding hip circumference on hip osteoarthritis.
