# Supplementary material for: Causal Relationships of General and Abdominal Adiposity on Osteoarthritis: A Two-Sample Mendelian Randomization Study
Source: J Clin Med. 2022 Dec 31;12(1):320. doi: 10.3390/jcm12010320 (PMC9820884; doi:10.3390/jcm12010320)
Supplement: Supplementary file 1 [file jcm-12-00320-s001.zip › Supplementary Figures.pdf]

Supplementary Figures

Figure S1 The MR results for causal effect regarding to waist circumference on knee osteoarthritis.

Figure S2 The MR results for causal effect regarding to waist circumference on hip osteoarthritis.

Figure S3 The MR results for causal effect regarding to hip circumference on knee osteoarthritis.

Figure S4 The MR results for causal effect regarding to hip circumference on hip osteoarthritis.

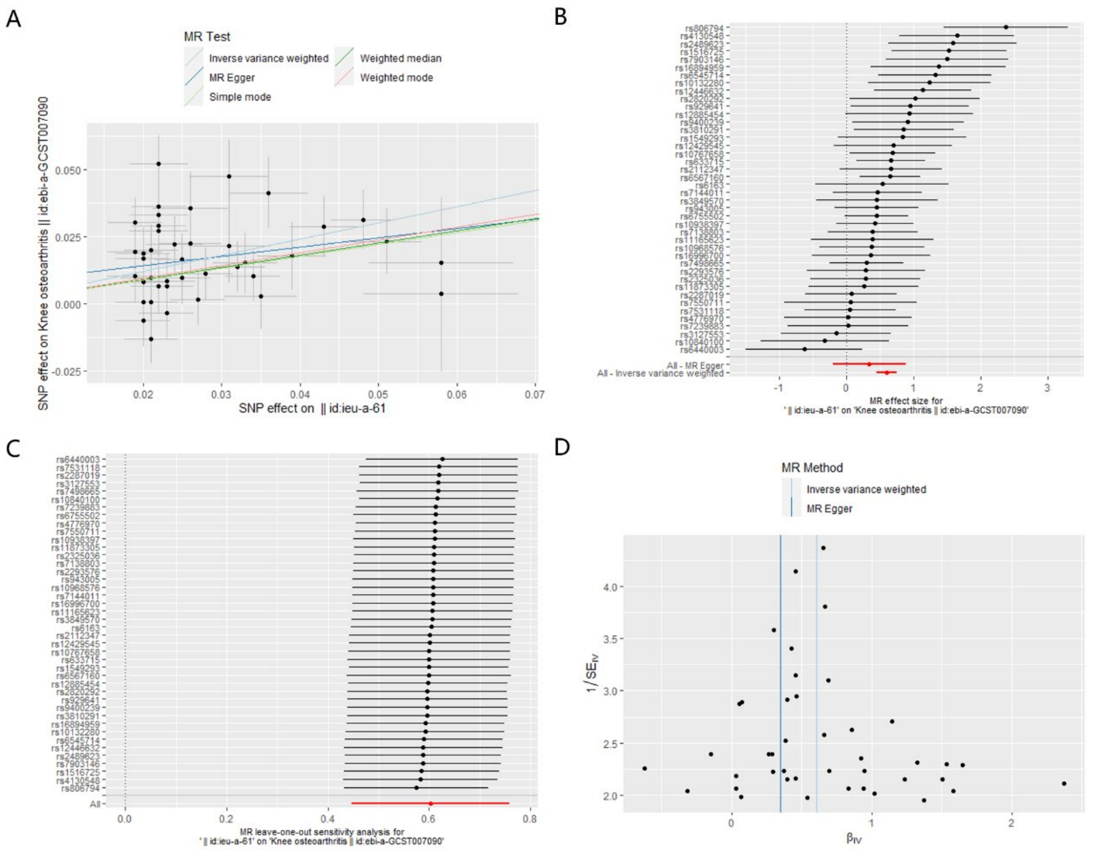

Figure S1 The MR results for causal effect regarding to waist circumference on knee osteoarthritis. A: Scatter plot about the casual effect of waist circumference on knee osteoarthritis; B: Forest plot for the overall causal effects of waist circumference on knee osteoarthritis; C: Leave-one-out analysis for the casual effect of waist circumference on knee osteoarthritis; D: Funnel plot of SNPs related to waist circumference on knee osteoarthritis.

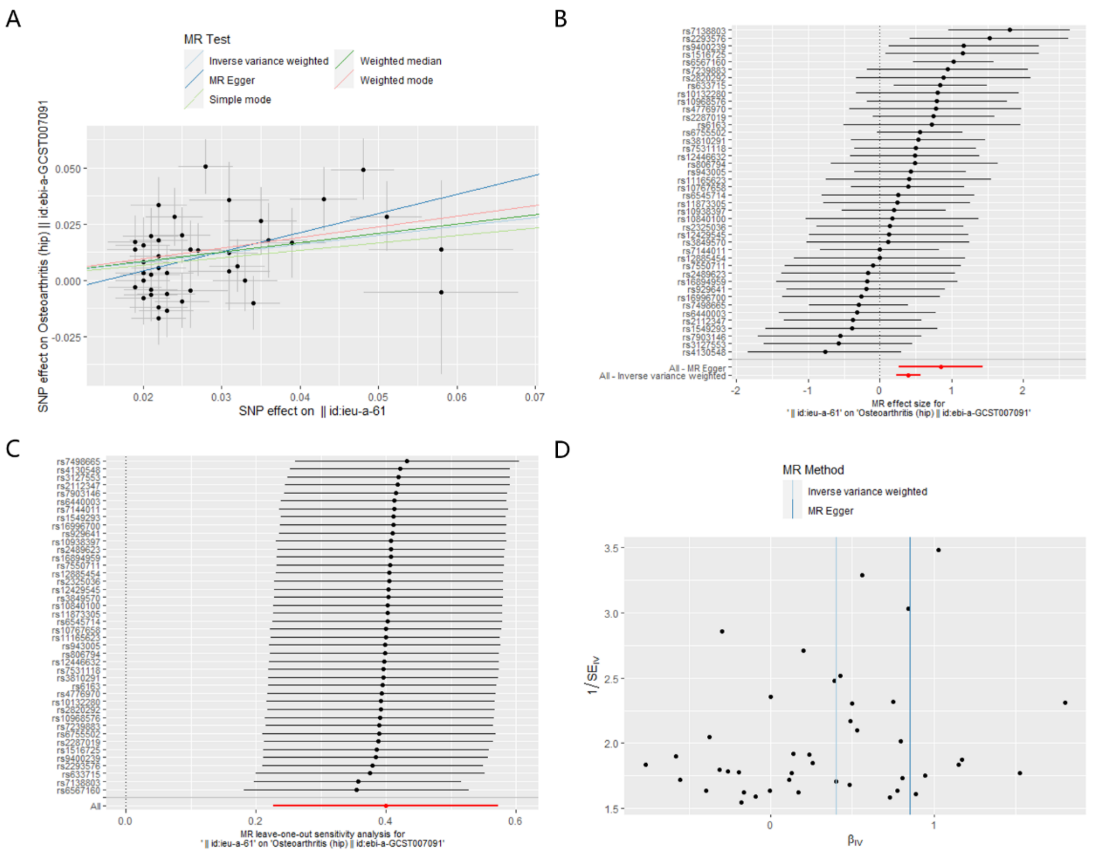

Figure S2 The MR results for causal effect regarding to waist circumference on hip osteoarthritis. A: Scatter plot about the casual effect of waist circumference on hip osteoarthritis; B: Forest plot for the overall causal effects of waist circumference on hip osteoarthritis; C: Leave-one-out analysis for the casual effect of waist circumference on hip osteoarthritis; D: Funnel plot of SNPs related to waist circumference on hip osteoarthritis.

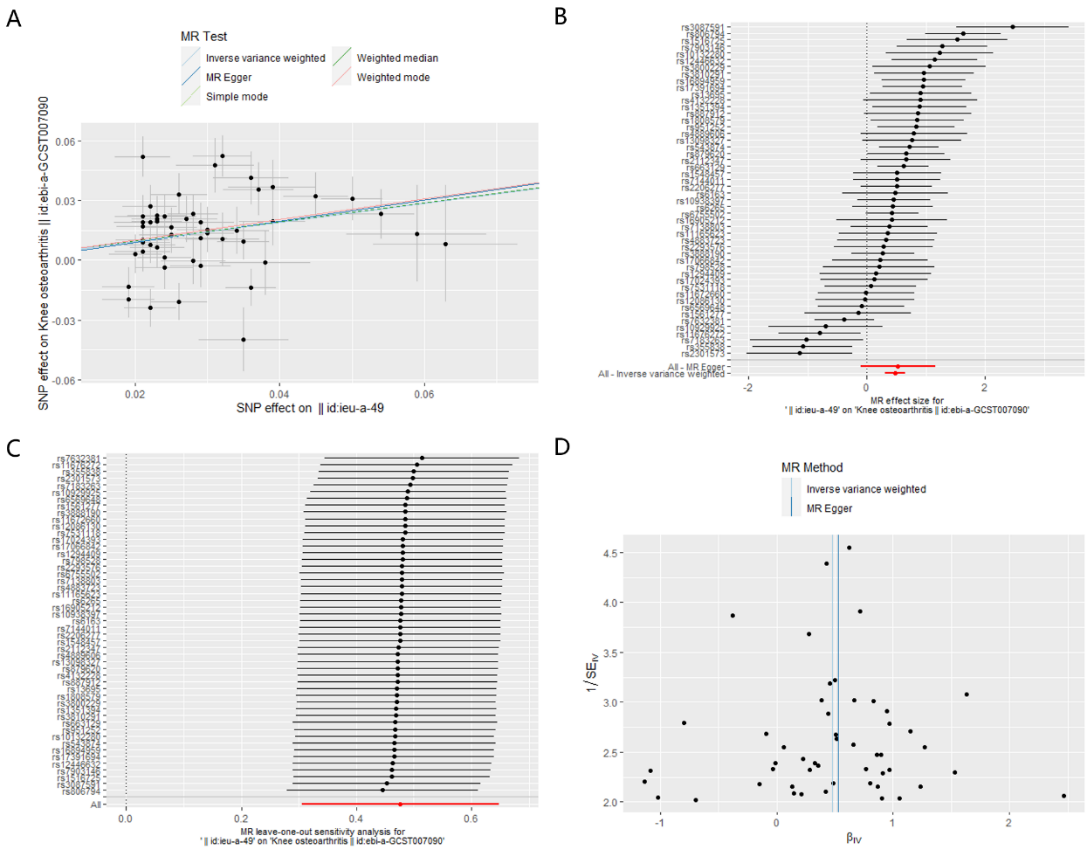

Figure S3 The MR results for causal effect regarding to hip circumference on knee osteoarthritis. A: Scatter plot about the casual effect of hip circumference on knee osteoarthritis; B: Forest plot for the overall causal effects of hip circumference on knee osteoarthritis; C: Leave-one-out analysis for the casual effect of hip circumference on knee osteoarthritis; D: Funnel plot of SNPs related to hip circumference on knee osteoarthritis.

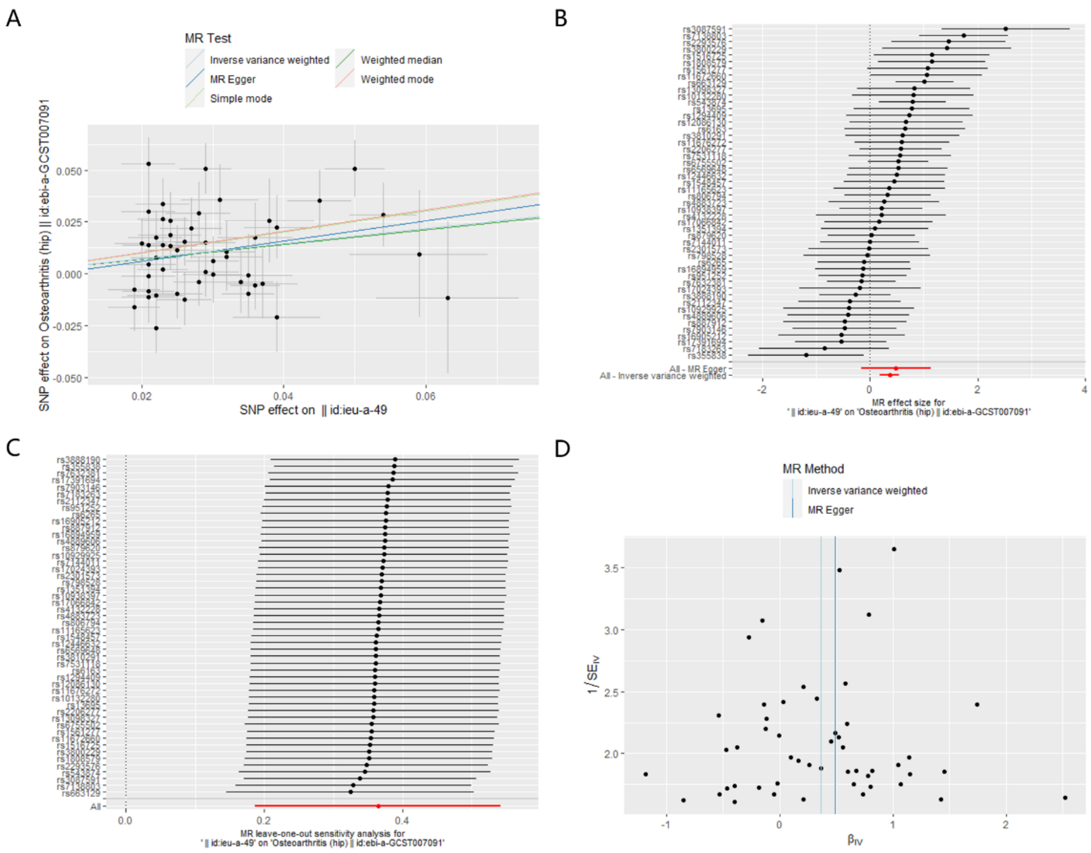

Figure S4 The MR results for causal effect regarding to hip circumference on hip osteoarthritis. A: Scatter plot about the casual effect of hip circumference on hip osteoarthritis; B: Forest plot for the overall causal effects of hip circumference on hip osteoarthritis; C: Leave-one-out analysis for the casual effect of hip circumference on hip osteoarthritis; D: Funnel plot of SNPs related to hip circumference on hip osteoarthritis.
